# Supplementary material for: In Silico Transcriptomic Expression of MSR1 in Solid Tumors Is Associated with Responses to Anti-PD1 and Anti-CTLA4 Therapies
Source: Int J Mol Sci. 2024 Apr 3;25(7):3987. doi: 10.3390/ijms25073987 (PMC11012116; doi:10.3390/ijms25073987)
Supplement: Supplementary file 1 [file ijms-25-03987-s001.zip › ijms-2926591-supplementary.pdf]

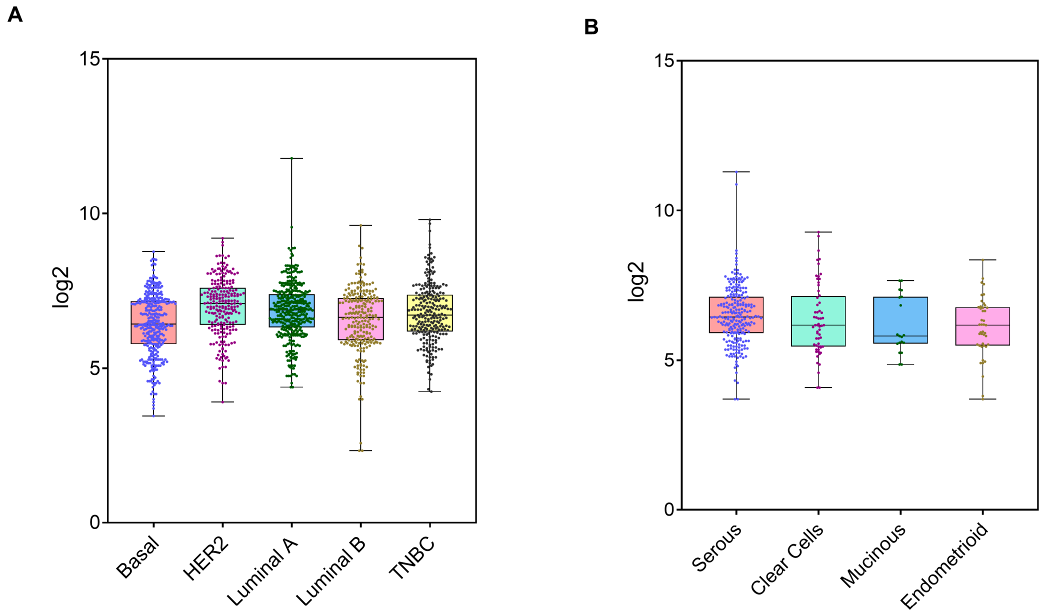

Supplementary Figure S1. Expression data obtained from GENT2. This web tool implies expression levels with the MAS5 algorithm which allows the comparison between independent datasets. a) *MSR1* expression data on breast cancer subtypes. B) *MSR1* expression data on ovarian cancer subtypes.

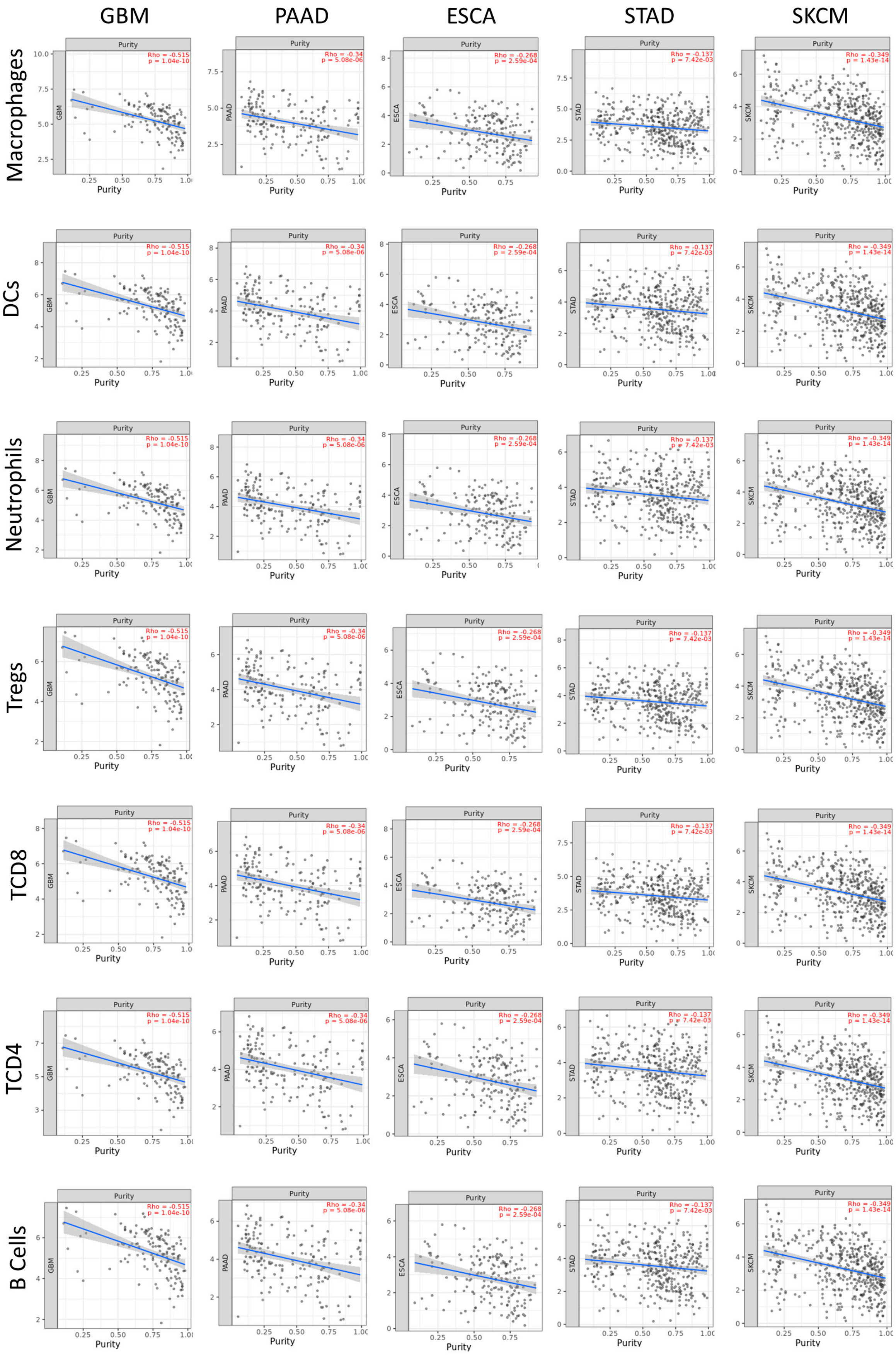
GBM
PAAD
ESCA
STAD
SKCM
Macrophages
DCs
Neutrophils
Tregs
TCD8
TCD4
B Cells
Purity
Rho = -0.515 p = 1.04e-10
Rho = -0.34 p = 5.08e-06
Rho = -0.268 p = 2.59e-04
Rho = -0.137 p = 7.42e-03
Rho = -0.349 p = 1.43e-14

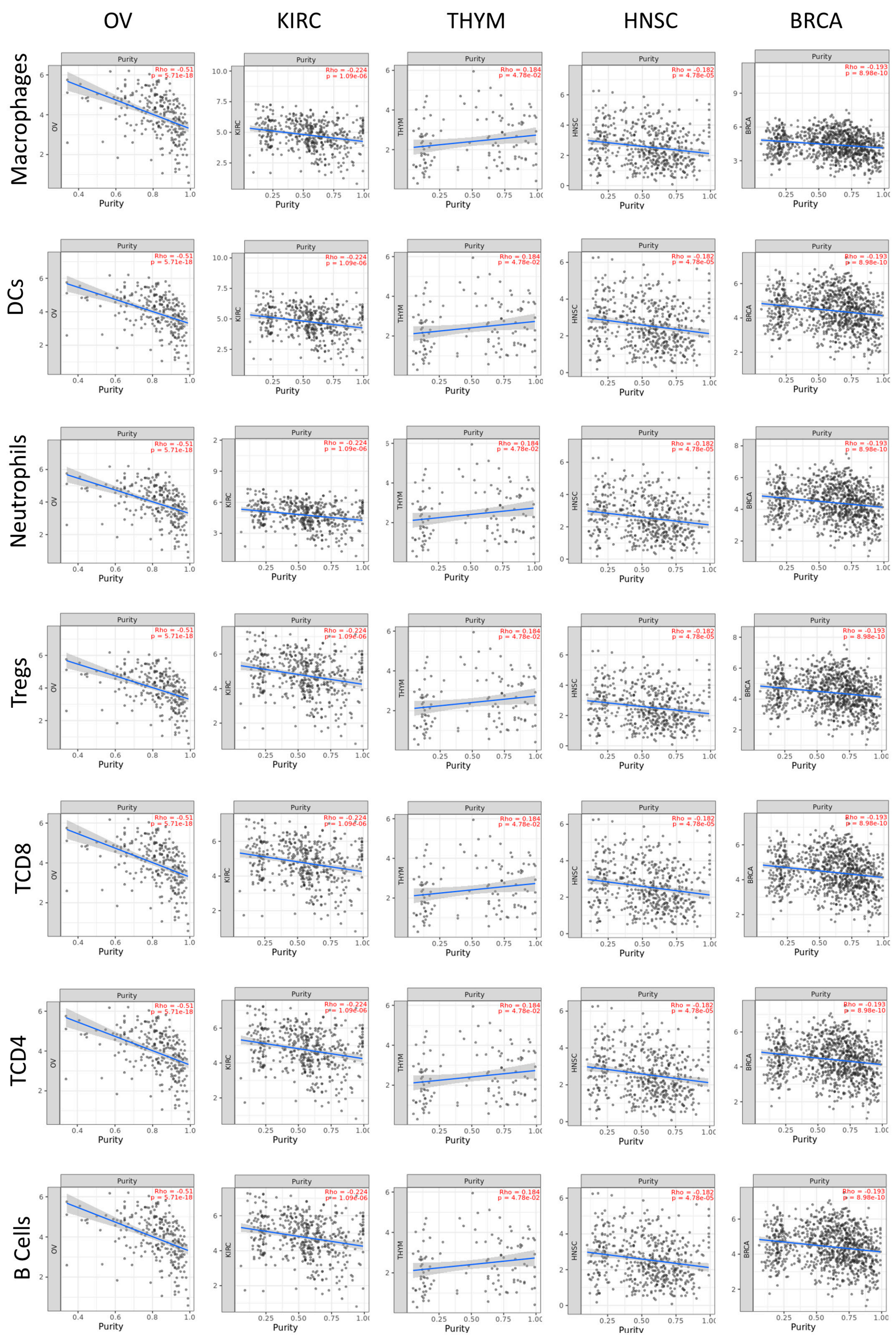

Supplementary Figure S2. **Dot plots for the purity in relation to the immune populations in the ten selected tumor types. *MSR1* expression is represented in the y axes as log2 (TPM) and purity level in the x axis.**

**Web Tool**	GEPIA2	GENT2	CBIOPORTAL	KM PLOTTER	TIMER2.0	ROC PLOTTER
**Data sets contained**	TCGA and GTEx	GEO	Several data sets	GEO, EGA andTCGA	TCGA	GEO, EGA andTCGA
**Link**	**https://www.cancer.gov/about-nci/organization/ccg/research/structural- genomics/tcga**<br><br>**https://gtexportal.org/home/**	**https://www.ncbi.nlm.nih.gov/geo/**	**https://www.cbioportal.org/datasets**	**https://www.ncbi.nlm.nih.gov/geo/**<br><br>**https://ega-archive.org/**<br><br>**https://www.cancer.gov/about-nci/organization/ccg/research/structural- genomics/tcga**	**https://www.cancer.gov/about-nci/organization/ccg/research/structural- genomics/tcga**	**https://www.ncbi.nlm.nih.gov/geo/**<br><br>**https://ega-archive.org/**<br><br>**https://www.cancer.gov/about-nci/organization/ccg/research/structural- genomics/tcga**

Supplementary Table S1. Table containing information about the data sets used and original source.
